# Acute Gastric Volvulus With Wandering Spleen in a Two-Year-Old Child: A Rare Association

**DOI:** 10.7759/cureus.38386

**Published:** 2023-05-01

**Authors:** Sunil Kumar Bhambu, Puneet Kumar Awasthi, Rahul Mangal, Ritu Mehta, Shreyas N

**Affiliations:** 1 Radiodiagnosis, Ananta Institute of Medical Sciences & Research Center, Udaipur, IND; 2 Surgery, Ananta Institute of Medical Sciences & Research Center, Udaipur, IND

**Keywords:** unusual association, life threatening, surgical emergency, wandering spleen, gastric volvulus

## Abstract

Gastric volvulus is a rare clinical condition characterized by a pathological rotation of the stomach greater than 180º around its axis. The wandering spleen is also an exceptional clinical entity characterized by the absence or laxity of splenic ligaments which lead to splenic mobility in the abdominal cavity from its normal anatomical site. Wandering spleen and gastric volvulus association is unusual. Both can be life-threatening if left untreated. We herein present a rare, unusual association of mesenteroaxial gastric volvulus with wandering spleen in a two-year-old child and interpret the radiological findings to ensure correct and early diagnosis.

## Introduction

Gastric volvulus is an abnormal rotation of the stomach greater than 180º around its axis - longitudinal or transverse axis [1]. Gastric volvulus is extremely rare and always a surgical emergency. Gastric volvulus is classified as either organoaxial, mesenteroaxial, or combined in relation to the torsion’s axis [2]. If left untreated it can be potentially life-threatening due to strangulation which can lead to ischemic necrosis and sometimes perforation if detected late. In such cases, the mortality rate varies from 30% to 50% [3]. A wandering spleen is characterized by excessive mobilization from the spleen’s normal anatomical location. The left upper abdominal quadrant is the most common abnormal anatomical position in the wandering spleen [4]. The pathogenesis is linked to either absence or laxity of ligaments responsible for keeping the spleen in place [4]. Association of gastric volvulus and wandering spleen is a rare condition, and around 52 cases have been reported till 2015, most of them are in children, and only three cases are in adults [5]. We are reporting this rare association in a two-year-old child presenting with intestinal obstruction.

## Case presentation

**Figure 1:**
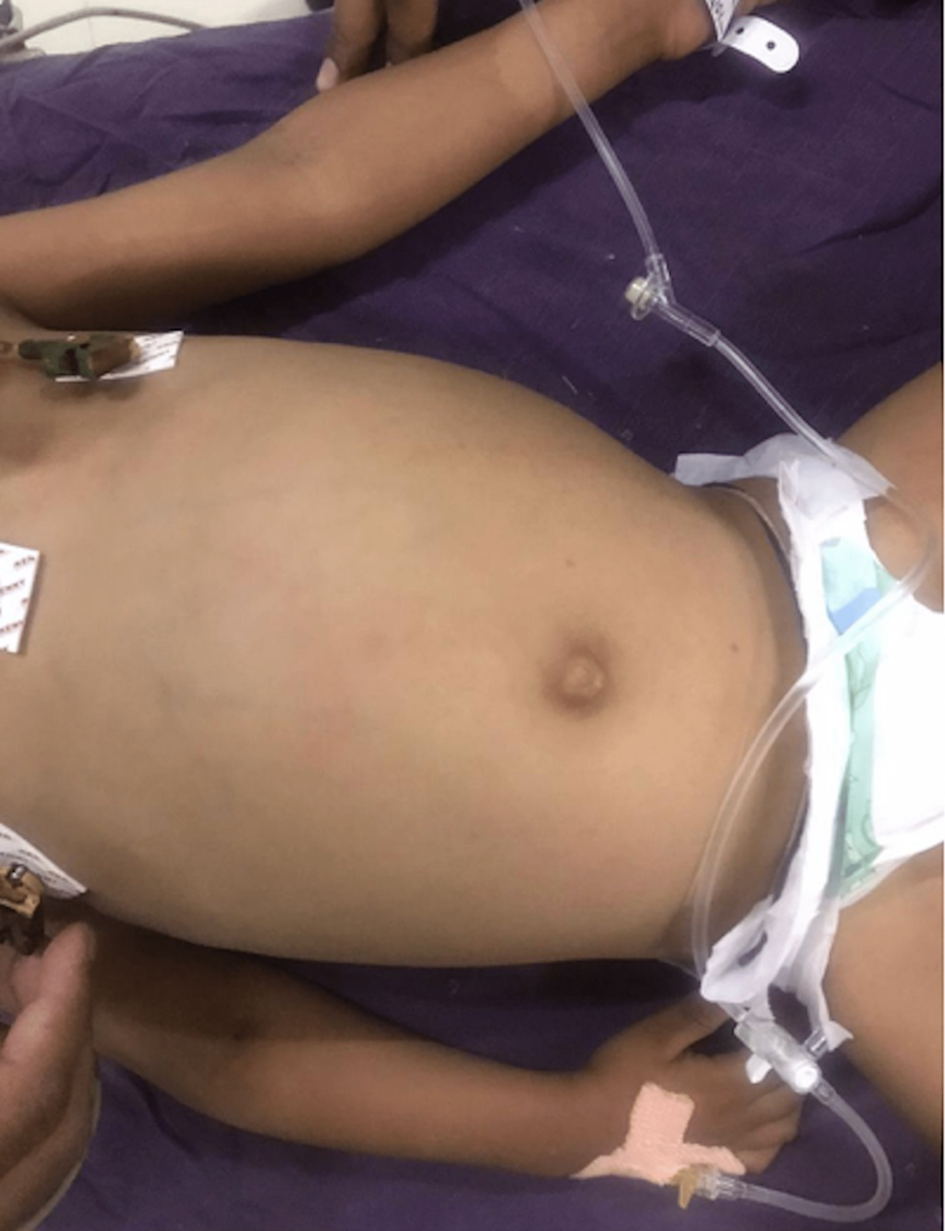
Distended abdomen

**Figure 2:**
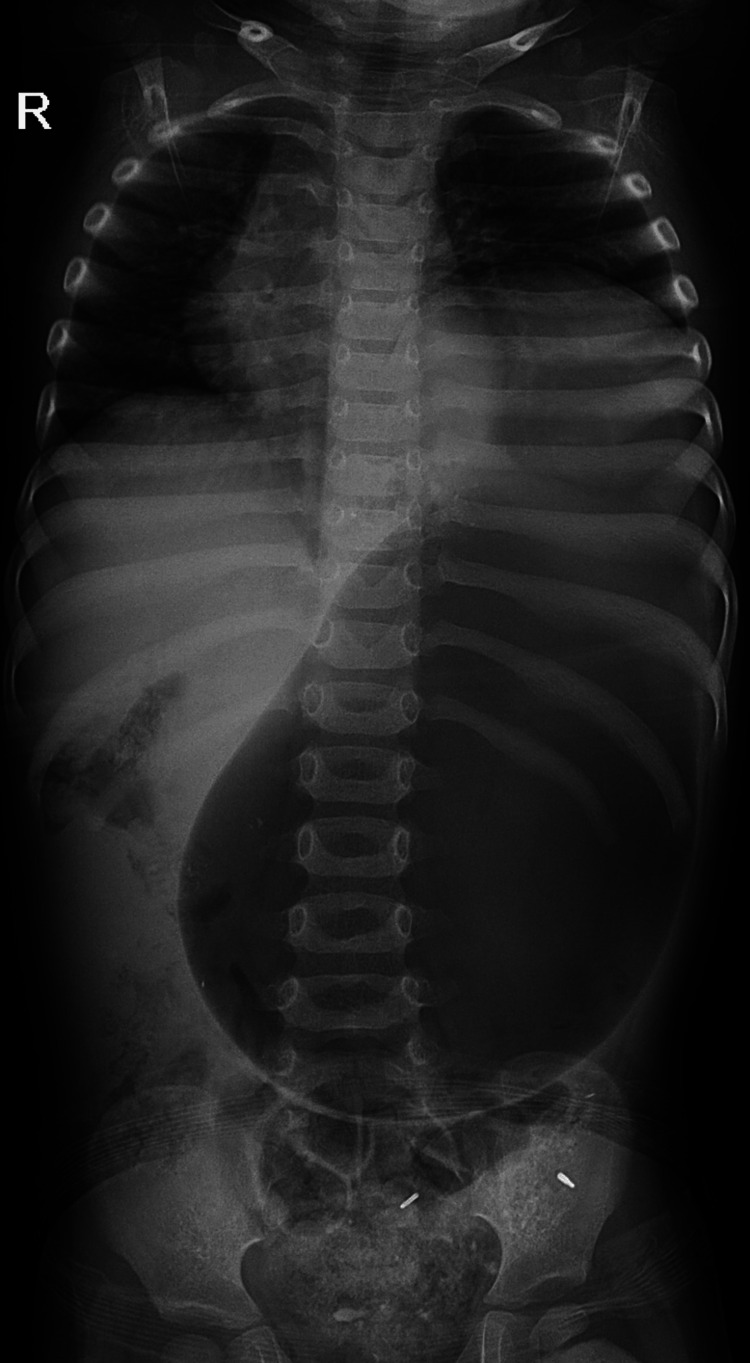
Flat plate (FP) abdominal skiagram showing elevated dome of left hemidiaphragm with distended gaseous mass/loop herniating into the left hemithorax.

A two-year-old male child was bought by his parents to the emergency department with complaints of abdominal pain, intractable retching with one episode of non-bilious vomiting for two to three hours, and a suspected history of foreign body ingestion. However, there was no episode of vomiting during a hospital stay. There was no history of fever, diarrhea, or trauma. On physical examination, the child was healthy, the pulse rate was 110/min, blood pressure was 114/66 mmHg, respiratory rate was 24/min and SpO_2_ was 99% at room air. The abdomen was tender and distended on systematic examination (Figure 1). However, other systematic examinations were unremarkable. Initial laboratory investigation (Complete blood count, liver and renal function tests) was within normal limits. Chest skiagram posteroanterior (PA) view and flat plate (FP) abdominal skiagram were done to rule out foreign body ingestion. The abdominal FP skiagram found an elevated left hemidiaphragm and a giant spherical radiolucent gaseous bubble in the left upper quadrant (Figure 2). However, bowel loops were not dilated, and no air-fluid levels or free gas were present in the abdominal cavity. Gastric volvulus was suspected on the basis of an abdominal skiagram. The child was initially managed with symptomatic intravenous therapy, and volvulus decompression by nasogastric tube insertion was planned. However, the nasogastric tube insertion attempt was unsuccessful. Subsequently, plain computed tomography (CT) of the abdomen was done to confirm the diagnosis.

**Figure 3:**
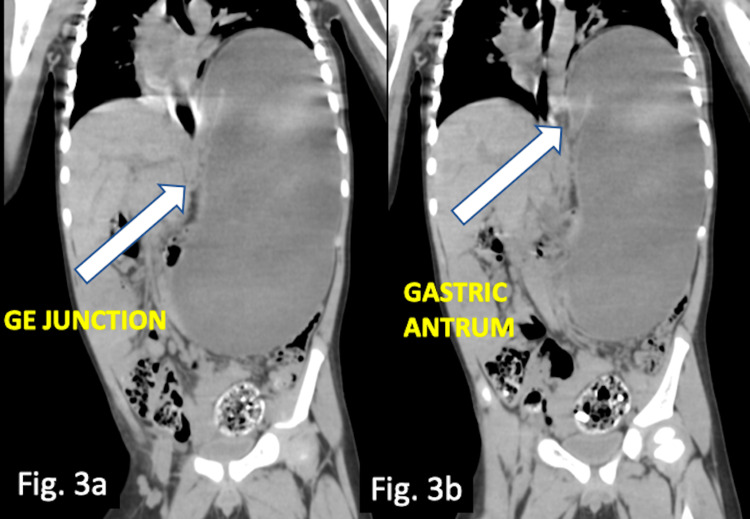
Non-contrast CT abdomen coronal sections showing (a) overdistended and (b) upside-down stomach.

**Figure 4:**
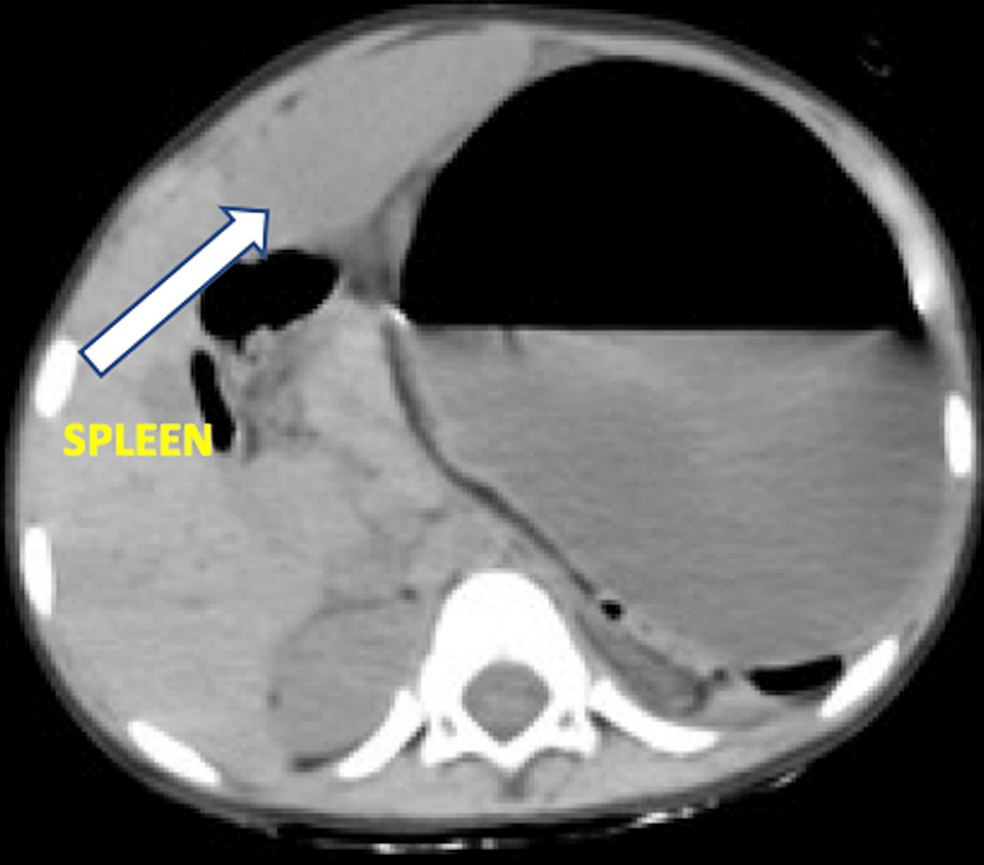
Non-contrast CT abdomen axial section showing paramidline right-sided spleen with overdistended stomach.

The CT scan findings showed overdistended stomach with high placed left dome of diaphragm. The antrum was displaced above the level of gastroesophageal junction, with upside down appearing stomach (Figures 3a, 3b). There was also displacement of spleen from its normal position into right paramidline position closely abutting the left lobe of liver (Figure 4). No diaphragmatic defect was noticed.

**Figure 5:**
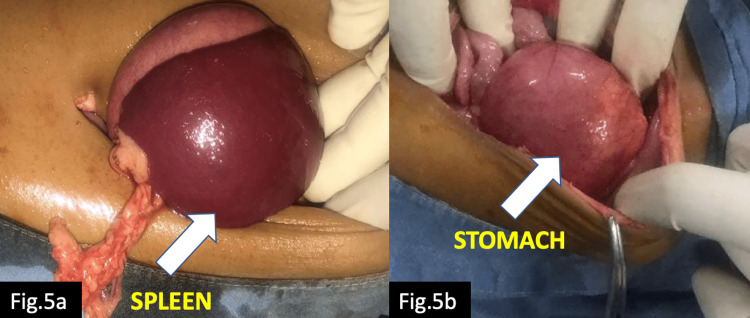
Intraoperative images showing displaced spleen on the right side (a) and distended stomach (b).

Pre-operatively, on the basis of clinical and radiological findings, diagnosis of mesenteroaxial gastric volvulus was confirmed. Under general anesthesia an urgent exploratory laparotomy was done. Intraoperatively, the stomach was found to be grossly distended and reaching to the pelvis. The greater curvature was not fixed on its entire length. The spleen was free floating in midline epigastrium towards right side (Figures 5a, 5b).

**Figure 6:**
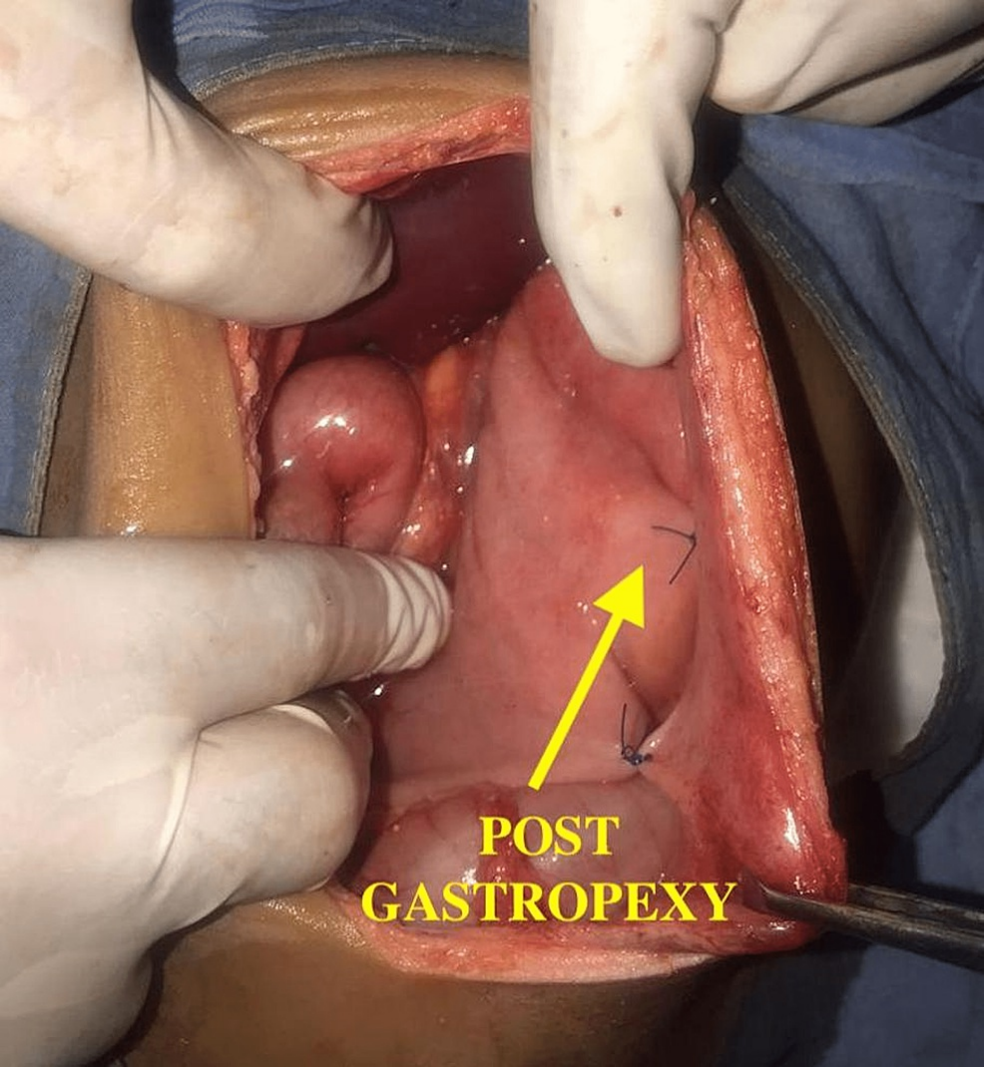
Intraoperative image showing post-anterior gastropexy.

**Figure 7:**
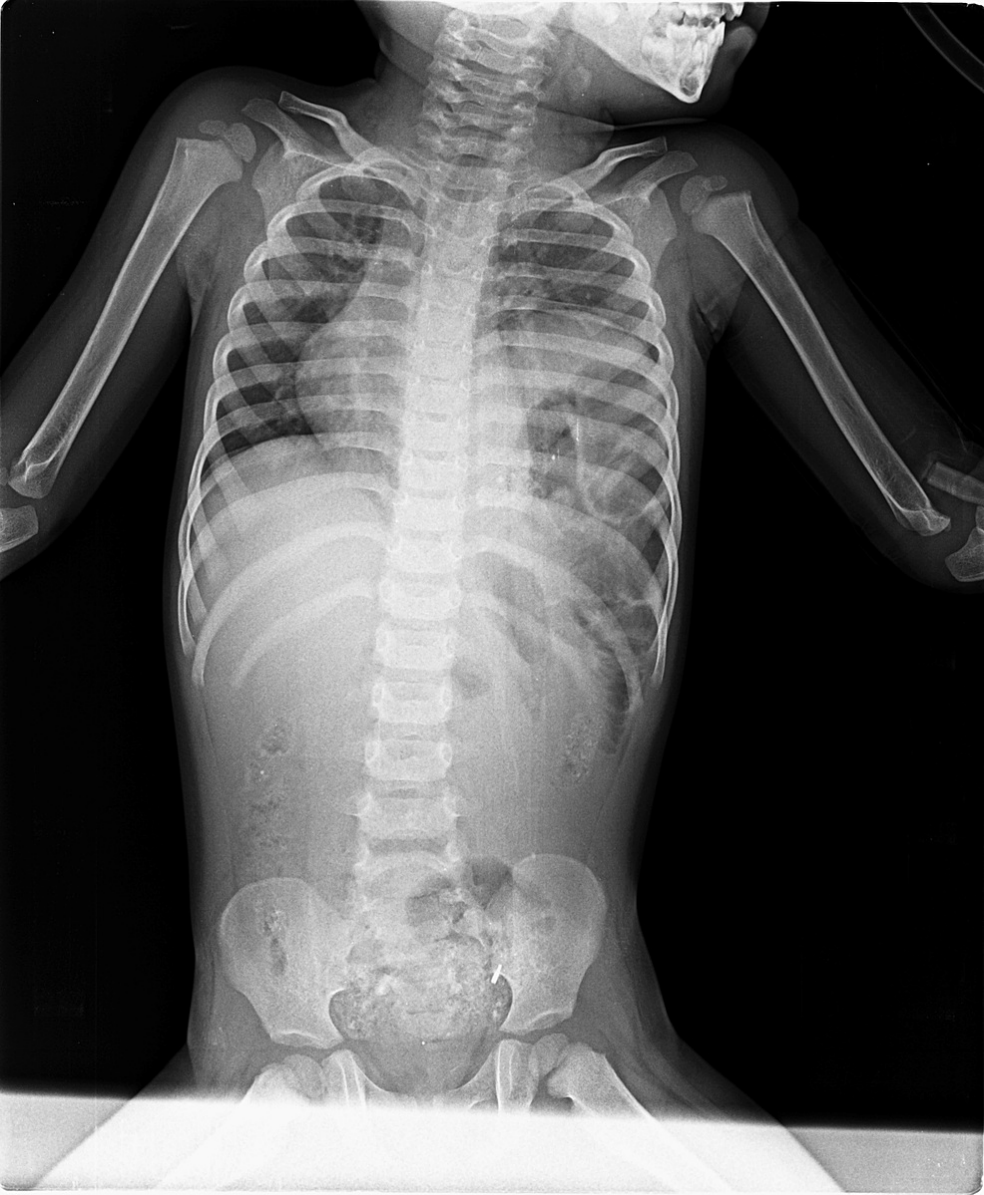
Post-operative day 3 flat plate (FP) abdominal skiagram showing almost complete resolution of giant spherical radiolucent gaseous bubble and elevated left hemidiaphragm.

After derotating, nasojejunal tube placed and decompression was done followed by anterior gastropexy and splenopexy (Figure 6). The FP abdominal skiagram done on day 3 of postoperative period shows almost complete resolution of giant spherical radiolucent gaseous bubble and elevated left hemidiaphragm (Figure 7). Postoperatively, the patient was discharged on day 7 and postoperative period was uneventful.

## Discussion

Gastric volvulus is the abnormal rotation of the stomach and commonly presents with symptoms of intestinal obstruction. The first case was described in pediatrics in 1899 by Oltmann; however, in adults, the first case was reported by Berti 33 years earlier in 1866. Moreover, in 1940 Singleton suggested the classification of gastric volvulus into three types on the basis of the rotation angle of the stomach axis [6]. First is organo axial, the most common type, presented in around 60% of cases. Second is mesentero axial, seen in around 26%, and third is mixed type, which is the least common [7]. This child had mesentero axial type of gastric volvulus. Though, mesentero axial volvulus is uncommon and a known surgical emergency, requiring early surgical intervention. However rare, if delayed in management, it can be associated with significant mortality. Nasogastric tube decompression was attempted but not successful in our patient also, and he underwent laparotomy followed by decompression with a nasojejunal tube and anterior gastropexy.

A wandering spleen is characterized by the absence of a spleen from its normal position, which can be present in any part of the abdomen. This condition is occurred due to the maldevelopment of visceral splenic ligaments. Moreover, the wandering spleen may be attached only to the vascular pedicle, and these vascular pedicle attachments have an increased risk of rotation which can lead to splenic congestion as well as infarction [8].

Though rare, the association of the gastric volvulus and the wandering spleen in a patient is due to typical developmental abnormalities in intraperitoneal visceral ligaments. All cases of gastric volvulus should be screened for wandering spleen preoperatively by ultrasonography, CT scan, and intraoperatively. Because, if left untreated further increases morbidity and mortality due to increased risk of congestion and infarction.

Common clinical features of gastric volvulus are related to gastric outlet obstruction like severe non-bilious vomiting, pain abdomen, and abdominal distention. These symptoms started mostly after acute rotation of the predisposed stomach. Borchardt’s triad included retching, epigastric swelling, and inability to pass the nasogastric tube, which is present in nearly 50% of patients with gastric volvulus [9]. This patient has the typical Borchardt triad at the time of presentation except for a history of one episode of non-bilious vomiting.

Gastric volvulus is not a common disease, but a plain radiograph of the chest and abdomen showing a distended stomach and gastric double bubble sign can lead to a diagnosis, however, the gold standard investigation is barium swallow and upper gastrointestinal (GI) contrast study [9]. In this case, an unexpectedly higher placed overdistended stomach with a paucity of small bowel air in the radiograph was suspicious, and later on, diagnosis of gastric volvulus was confirmed on CT scan.

Surgical correction either open or laparoscopic is required for the management of gastric volvulus. Surgical decompression of the stomach and restoration of the angle of rotation with gastropexy is the procedure of choice. Splenopexy should be done if associated with a wandering spleen otherwise this may further complicate the situation with torsion. This patient underwent open laparotomy, gastropexy, and splenopexy within four hours of admission. The postoperative period was uneventful and discharged with follow-up advice in the surgical outpatient department (OPD).

## Conclusions

The child presented with abdominal pain and vomiting with suspicious history of foreign body ingestion. He had no past history of diaphragmatic defects. So, on initial presentation, the diagnosis of acute gastric volvulus is tough but with the help of classic imaging findings on plain radiographs we diagnosed, treated the patient immediately within four hours of presentation, and prevented complications.

Gastric volvulus with wandering spleen is a rare condition and a high index of suspicion on a plain radiograph can clinch the diagnosis. Though rare if untreated gastric volvulus and wandering spleen are associated with high mortality. Immediate surgical decompression and restoration of rotation is the procedure of choice.
